# Global Healthy and Sustainable City Indicators: Collaborative development of an open science toolkit for calculating and reporting on urban indicators internationally

**DOI:** 10.1177/23998083241292102

**Published:** 2024-12-02

**Authors:** Carl Higgs, Melanie Lowe, Billie Giles-Corti, Geoff Boeing, Xavier Delclòs-Alió, Anna Puig-Ribera, Deepti Adlakha, Shiqin Liu, Júlio Celso Borello Vargas, Marianela Castillo-Riquelme, Afshin Jafari, Javier Molina-García, Vuokko Heikinheimo, Ana Queralt, Ester Cerin, Eugen Resendiz, Dhirendra Singh, Sebastian Rodriguez, Esra Suel, Marc Domínguez-Mallafré, Yang Ye, Amanda Alderton

**Affiliations:** 5376 RMIT University, Australia; 5376 RMIT University, Australia; 5376 RMIT University and The University of Western Australia, Australia; 5116 University of Southern California, USA; 16777 Universitat Rovira i Virgili, Spain; 16783 University of Vic-Central University of Catalonia, Spain; Delft University of Technology, The Netherlands; 5635 University of Minnesota, USA; 28124 Federal University of Rio Grande do Sul, Brazil; 14655 Universidad de Chile, Chile; 5376 RMIT University, Australia; University of Valencia, Spain; 73144 Finnish Environment Institute, Finland; University of Valencia, Spain; 95359 Australian Catholic University, Australia; 12330 The University of Texas at Austin, USA; 170512 CSIRO’s Data61, Australia; 5376 RMIT University, Australia; 4919 University College London, UK; 16777 Universitat Rovira I Virgili, Spain; 47822 Harbin Institute of Technology, China; 5376 RMIT University, Australia

**Keywords:** Software development, open science, healthy cities, sustainability, city planning, urban indicators, urban policy, spatial analysis, research translation, action research

## Abstract

Measuring and monitoring progress towards achieving healthy, equitable and sustainable cities is a priority for planners, policymakers and researchers in diverse contexts globally. Yet data collection, analysis, visualisation and reporting on policy and spatial indicators involve specialised knowledge, skills, and collaboration across disciplines. Integrated open-source tools for calculating and communicating urban indicators for diverse urban contexts are needed, which provide the multiple streams of evidence required to influence policy agendas and enable local changes towards healthier and more sustainable cities. This paper reports on the development of open-source software for planning, analysis and generation of data, maps and reports on policy and spatial indicators of urban design and transport features for healthy and sustainable cities. We engaged a collaborative network of researchers and practitioners from diverse geographic contexts through an online survey and workshops, to understand and progressively meet their requirements for policy and spatial indicators. We outline our framework for action research-informed open-source software development and discuss benefits and challenges of this approach. The resulting Global Healthy and Sustainable City Indicators software is designed to meet the needs of researchers, planners, policy makers and community advocates in diverse settings for planning, calculating and disseminating policy and spatial urban indicators.

## Introduction

An increasing majority of the world’s population resides in cities, but inequities in access to employment, services and amenities persist (United Nations, 2020). Poor urban design exacerbates inequities, posing challenges for population health and planetary sustainability (Seto et al., 2017). Evidence-informed urban policy and spatial indicators can support integrated city planning at all levels of government, by identifying whether and where urban policies for healthy and sustainable cities are being delivered (U. N. Habitat and World Health Organization, 2020). Measurement, analysis and monitoring of indicators within cities across time can help ensure planning priorities are being delivered effectively and equitably (Geurs and Van Wee, 2004; Karner et al., 2024). However, to make a difference, relevant evidence needs to reach actors across the multiple sectors involved in shaping urban environments (Lowe et al., 2019). Community advocates, urban and transport planners, policymakers and public health researchers require accessible, locally contextualised urban indicators calculated at a range of scales to enable within- and between-city comparisons (Giles-Corti et al., 2022): policy indicators to evaluate policies that govern and regulate urban development; as well as spatial indicators for assessing whether, where and for whom urban design and transport features have been delivered.

Advances in computational power and data availability mean that there is unprecedented technical capacity to identify where policies and planning goals are not being met and inform interventions to improve urban equity for diverse contexts globally (Boeing et al., 2022; Kang et al., 2019). However, the effective realisation of urban indicators to support planning for healthy and sustainable cities – indicators that are comparable, contextualised, meaningful and reproducible – is hindered by a concurrent crisis in reproducibility and trust in science (Essawy et al., 2020; Peng, 2015; Wolf, 2023). Scientific publications endorse ideals of open science (UNESCO, 2021) and FAIR research (Wilkinson et al., 2016) by encouraging publication of code that can be reviewed, and in principle used to produce and reproduce analyses (Arribas-Bel et al., 2021; Essawy et al., 2020). A further barrier to indicators informing decision making is that the availability of relevant, high-quality data is unevenly distributed globally and within cities, and therefore must be approached critically. In the words of Sharma et al. (2023), ‘*Data cannot be decoupled from the biases of those who create, collect, and analyze them*’. For local impact, the planning, design, conduct and publication of analyses are best undertaken by or with the involvement of locally connected stakeholders (Helm et al., 2023), who are motivated to share new urban indicator evidence with their network and advocate for the healthy and sustainable city policies that evidence supports (Giles-Corti et al., 2022).

The pursuit of open science standards in locally engaged urban science is an ongoing challenge (Wolf, 2023). Wilson et al. (2021) note that a research culture shift is required to achieve a ‘five-star rating’ for reproducibility and replicability. There is a lot of specialist expertise involved to achieve meaningful and repeatable analyses with output reporting that is useful and engaging for relevant stakeholders. Effective modelling and reporting on the complexity of urban system demands interdisciplinary expertise (Black et al., 2018), spanning diverse fields: urban planning, population health, spatial and network analysis, statistics, visual communication design and research translation. Moreover, to ensure reproducibility, proficiency in information management, programming and scientific communication is essential.

While there are other global indicator frameworks (U. N. Habitat and World Health Organization, 2020), there is a notable gap in the availability of tools that align local audits of healthy cities policies with high-resolution spatial indicators of their delivery in data, figures and reports that are meaningful and accessible to a wide range of stakeholders. This gap is particularly evident in the context of diverse urban environments, where local nuances and varying data availability can significantly impact analysis and interpretation. This research addresses this gap in provision of an integrated, open-source tool that allows local users to calculate urban indicators for their cities and generate the multiple streams of evidence required to influence policy agendas and enable local changes towards healthier and more sustainable cities (Kingdon, 1993; Rawat and Morris, 2016).

## Methods

Our project builds on a series of Python scripts comprising an open-source configurable spatial indicator workflow (Liu et al., 2021). This was developed for a proof-of-concept analysis of policy and spatial indicators for 25 cities across 19 countries and 6 continents (Boeing et al., 2022; Lowe et al., 2022). Measures of walkable neighbourhoods were calculated for point locations, 100 metre population-grids and overall city estimates in defined urban study regions. We used open data products with global scope, such as OpenStreetMap (OpenStreetMap contributors, 2023), the Global Human Settlements Layer (European Commission, 2023) or custom data where required. We validated indicator outputs with local research collaborators, including a walkability index, population and intersection density metrics and the percentage of population with access to fresh food, regularly serviced public transport and large public open space (Boeing et al., 2022). A separate audit of urban and transport planning policies was used to derive scores for presence and quality of policies for achieving healthy and sustainable cities (Lowe et al., 2022). In coordination with local collaborators from the study’s global research network, additional software was developed to generate policy and indicator reports based on a standard template, that could be customised for each city and present results using verified translations for local audiences (Higgs, 2022; Higgs et al., 2022), and subsequently used in local launches and town hall events (Higgs et al., 2023a, 2023b).

### Action research-informed software development

**Figure 1.**
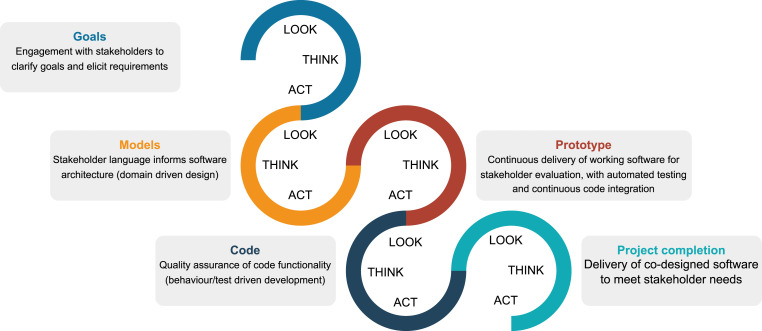
Our diagram illustrating the fusion of action research and software engineering as an iterative, cyclical process of improvement towards working software that meets stakeholders’ evolving needs.

Leveraging insights from the background research, we further developed a framework (Figure 1) fusing action research (Stringer and Aragón, 2021) with modern software engineering practices (Beck et al., 2001; Evans, 2003; Farley, 2022) in order to develop the existing workflow into an integrated open-source software toolkit for urban indicator calculating and reporting. Our motivation was to support local city teams participating in a ‘1000 City Challenge’ to measure and monitor healthy and sustainable urban design and planning across cities in diverse settings worldwide (Global Healthy and Sustainable City Indicators Collaboration, 2022). Through this framework, we proposed to engage domain expert urban planning/policy practitioners and researchers from diverse urban settings to build knowledge of their requirements for measuring, evaluating and reporting on policy and spatial indicators for positive local impact.

Action research is a participatory research methodology concerned with delivering tangible outcomes that address real-world problems (Stringer and Aragón, 2021). It aligns with the values and principles of the Agile manifesto for software development (Beck et al., 2001), a method for iterative delivery of functional software that meets the needs of users through reflection, adjustment and evaluation. Action research and Agile principles both emphasise the role of respectful collaboration to better understand a community’s requirements. We developed the diagram in Figure 1 to illustrate how building shared knowledge of a project’s goals facilitates their realisation through iterative and incremental progress. Participant ‘co-researchers’ led the development of working software through iterative cycles of observing, thinking and acting, resulting in progressive refinement of prototypes to meet stakeholders’ urban indicator reporting needs.

### Recruitment and participation

We identified and engaged three target groups in a practitioner-led research programme to determine their urban indicator software and software-output needs and to evaluate and refine their implementation: 1) those interested in participating in the 1000 Cities Challenge as representatives for diverse cities internationally; 2) members of the Observatory executive, representing the overall vision for the 1000 Cities Challenge; and 3) others who expressed interest in calculating and reporting on urban indicators using our framework. Participants were engaged as co-researchers, with shared ownership of the process and outputs, and invited to contribute as co-authors on this paper describing the co-developed software.

Human research ethical approval was granted by the RMIT University Design and Social Context (DSC) College Human Advisory Network (project ID 25552). Study information was shared via https://healthysustainablecities.github.io/software_feedback. The approved processes for recruitment, feedback sessions, requirement analysis and software development are further detailed in Supplementary Material, along with a completed Standards for Reporting Qualitative Research guidelines checklist (O’Brien et al., 2014).

Full details of the ethical-approved study methods are provided in Supplementary Material. Briefly, participants initially contacted in March 2023 were invited to complete an online survey and participate in a series of one-hour workshops, respectively, focused on use of the software and reporting requirements. Descriptive analysis of survey data was conducted using Python (Van Rossum and Drake, 2009) and is detailed in Supplementary Material. Code and anonymised data on user requirements have been published in conjunction with this paper. Workshops were conducted online via Microsoft Teams, repeated across different time zones, video-recorded and auto-transcribed. Following each of the workshops, video recordings were shared with co-researchers along with thematic analysis summaries of the points raised via e-mail and on the GitHub software repository discussion board. Co-researchers had the opportunity to amend their recorded suggestions as required and further engage with software development via e-mail and GitHub to ensure their specific needs were progressively met.

## Results

Across the three invited target groups (*n* = 39), 20 co-researchers (51.3%) completed the survey and/or participated in the formally organised workshops (Table 1). Co-researchers further supported the software development process through ad hoc engagement via e-mail conversations, online meetings and via the GitHub software development platform between March and October 2023. Supplemental Table S2 summarises the backgrounds, experience and requirements of co-researchers. A total of 17 co-researchers completed the online enrolment survey (Table 1); approximately half (9/17; 47%) were early adopters, six (35%) were from the Observatory executive team, and three (18%) were practitioners/researchers in fields related to urban planning and health subsequently recruited through interest in the project. Most co-researchers (13/17; 76%) had spent 6 or more years in their profession, and some reported having multiple roles. The cohort was highly educated with all having completed tertiary education and most having a PhD degree (14/17; 82%). Approximately half the cohort identified as female (8/16; 50% of responders). Co-researchers’ ages ranged between 20 and 60 years, with most in their 30s or 40s (13/17; 76%). Co-researchers’ primary location of work was situated in 13 cities across 9 countries, spanning Europe, North and South America, Oceania and Asia. In addition to English, more than half of co-researchers (*n* = 9) used other languages as part of their professional practice.

**Table 1.** Research participation of individuals involved in urban planning and health-related fields, by group.

Group	Participated / Invited (%)		
	Survey	Workshops	Overall
1000 Cities *C*hallenge early adopter	8/9 (88.9)	9/9 (100.0)	9/9 (100)
Observatory executive	6/14 (42.9)	5/14 (35.7)	8/14 (57.1)
Other policymaker/planning/research practitioners	3/16 (18.8)	1/1 (100.0)	3/16 (18.8)
Total	17/39 (43.6)	15/24 (62.5)	20/39 (51.3)

Survey results are reported in detail in Supplementary Material and summarised here. Co-researchers reported a mixed level of experience with spatial or policy analysis, using or publishing open data, and stakeholder engagement (Figure S3). They envisaged a broad cross-disciplinary audience for the proposed software toolkit (Table S4), with outputs ideally including documented data, maps, reports and comparisons with capacity for local contextualisation (Table S5). Co-researchers working in languages other than English valued support for indicator reporting in alternate languages highly (Figure S4). Most users preferred to run the software via a website, as a Python or R software module, or as a ‘point and click’ desktop application (Figure S5). Of the 24 co-researchers directly invited to attend the initial ‘process’ and ‘reporting’ workshop feedback sessions, 15 attended at least one workshop (Table 1), including the lead author as a participant-observer in all workshops (Robey and Taylor, 2018), moderating each of the sessions and participating in discussions. Consolidated feedback summaries of user feedback are included in Tables S7–S15, grouped by topic and aligned with actions undertaken.

### Running the software

Co-researchers emphasised that the software should be easy to use for applied practitioners with non-technical backgrounds. Early in the first workshop, a researcher with an urban health policy background asked,

Are you thinking of making this tool so easy to use that people that are not specialized and could [be] working in city councils or super municipality organizations could use it or are we still thinking of a tool that needs an expert to run it?

It was recommended (Supplemental Table S7) to make the software ‘*so easy to use that applied users who are not technical specialists could use it; under the hood it may be complex, but* [it’s] *important to not make it too complicated to use*’.

Co-researchers acknowledged the trade-offs between ease and flexibility; once more customisable options are provided, things become inherently more complicated. One participant specifically requested support for use as a Python library that could be run interactive code notebooks. However, another commented on lack of familiarity with open-source programming languages in their disciplinary context: ‘*Geospatial analysis is very popular in urban planning and architecture research, but…* [most researchers] *cannot do the coding*’. It was suggested that providing code examples would lower barriers to conducting reproducible research.

Ultimately we implemented four modes of usage for analysing configured urban study regions and generating reports and resources using the installed software: via a web browser, through a user-friendly app (Supplemental Figure S2); as command-line prompts (‘configure’, ‘analysis’, ‘generate’ and ‘compare’); as a Python module for advanced flexibility; or within the provided JupyterLab app (https://jupyter.org). A fully annotated example interactive Python notebook illustrating core functionality demonstrated the latter approach. The web application’s graphical user interface supports selecting and initialising new city configurations, running analyses, mapping indicators, generating reports, viewing study region comparisons and summarising completed policy checklists. Additionally, a function to export interactive HTML choropleth maps for exploring the spatial distribution of indicator results at a range of scales was incorporated and demonstrated in the provided Jupyter notebook example and instructional video.

### Configuration and customisation

Those with geospatial experience indicated that the then-current form of the software was ‘*easy*’, except for the configuration process, which was described as ‘*confusing*’ and ‘*complicated*’ (Supplemental Table S7). Regions were configured using structured text configuration files. These were intended to be readable for both humans and machines and support version control to enhance reproducibility and collaboration. However, with human users struggling to understand the approach to configuration, misconfiguration could occur, with analysis unable to proceed until the causes of errors were identified and overcome. More guidance and examples were requested. The use of one long file containing examples for all cities was also described as confusing. While these examples were intended to demonstrate flexibility for representation of different geographic contexts, co-researchers reported that the structure was overwhelming.

Responding to feedback, we introduced city-specific configuration files. Users could modify the example city (Las Palmas de Gran Canaria, Spain) to refer to data, attributions and parameters for their own city. We provided detailed comments to guide users on how to interpret and modify parameters, analyse and report on their own study regions.

Co-researchers requested features to support customisation for their own city context and practice (Supplemental Table S8). One co-researcher noted that OpenStreetMap data can be problematic for some contexts: ‘*For bigger cities yes, it works, but for the city I come from, which is 600,000 people, data is very scarce still, so the result is affected by that*’. The co-researcher posed the question, ‘*Is there a way to incentivise a growth of open data?’*. To address such limitations, the capacity to configure alternative points of interest data had been incorporated in the earlier prototype workflow (Liu et al., 2021). Responding to co-researcher feedback, we extended functionality to optionally configure official or custom street intersection data where available and where this provided a more accurate representation of street connectivity.

Co-researchers also requested customisation for population data. For example, the modelled, high-resolution, multi-temporal population data published as part of the Global Human Settlements Layer (Schiavina et al., 2019) are useful for comparable analyses across multiple cities but may provide inaccurate population estimates in some urban contexts. Such issues may be identified during the validation stage of analysis, and responding to user requests we incorporated functionality to optionally use alternative data, such as a country’s official population grid. Additionally, some participants requested the option to use specialised population datasets to report on indicators specific to demographic sub-groups. Working with Spanish co-researchers, data published for Catalonia, Spain, on total population stratified by gender and age was used to prototype functionality and demonstrate feasibility of using small area vector population datasets at relevant scales such as census tracts as an alternative to raster population data for the city of Tarragona. When working with vector data, a user could specify a column to be used for the population sub-group of interest as well as a denominator for total population to be used when calculating neighbourhood indicators related to population density.

### Comparing indicators within- and between cities

Co-researchers emphasised the need to conduct comparisons with contextually relevant peer cities to meaningfully engage with stakeholders, in particular by those working professionally in languages other than English (Supplemental Table S14). Some noted that different cities may require different reference standards, to ensure fair comparisons. For example, compact cities in Europe were said to represent ‘*a different reality*’ compared with many cities in low- and middle-income countries, but also the low-density sprawling urban forms typical of Australian and US cities.

Conversely, two Observatory executive members were concerned that software users’ self-selected comparison cities or thresholds could lead to low ambition: one suggested basing comparisons on best-practice evidence, to push ambition; the other suggested removing between-city comparisons altogether to avoid subjective interpretations or poor comparisons. While one non-executive group member suggested performance ranking of districts and neighbourhoods, an Observatory executive participant argued that while comparisons are very important, they should not be presented as a league table, as explicit ranking could deter city participation. An agreed solution was to support users to data, maps and reports detailing within- and between-city comparisons for their regions of interest, while the official reports endorsed by the Observatory and 1000 Cities Challenge could employ criteria, comparisons and formatting determined by the Observatory executive in consultation with the local teams who did the analysis. It was observed that, in the future, results from the cities that have participated in the 1000 Cities Challenge could provide informative benchmarks that could be used for relevant peer-city comparisons.

Four co-researchers emphasised the need for longitudinal comparisons within cities to evaluate the impact of urban actions over time. One explained,

If we could use these spatial analyses for a very specific neighbourhood … before the urban intervention and after the intervention, I think this would be very useful for urban planners, municipalities and public health practitioners… [Because] whenever we want to evaluate the health impact of any...urban actions, it’s something difficult to do… this is what [decision-makers] demand … it would offer them a tool that doesn’t exist at the moment.

We incorporated a ‘compare’ function to streamline generation of side-by-side comparisons of spatial indicators – displayed on screen and exported as CSV files – for different configured study regions that have been analysed with resources generated. This could support analyses meeting some of the participants’ requests:

• evaluate the overall impact of parameters and data used (sensitivity analyses);
• compare results of different cities (benchmarking);
• compare results for the same study region across time (monitoring);
• evaluate impacts of hypothetical scenarios or interventions through analysis of modified data.

The compare tool allows sensitivity analyses to be performed to evaluate how methodological decisions, such as the way in which a study region boundary has been defined, can influence spatial urban indicator results. The impact of this choice should be understood and, depending on context and the needs of those who would use the indicator data and estimates produced, customisation of boundary definition may be required. Some co-researchers explained that administrative boundaries (e.g. local government boundaries) could be preferable for communicating with local policymakers about interventions within a specific city, district or neighbourhood. We developed guided examples on how to conduct a basic sensitivity analysis and evaluate how a change in study region definition can impact indicator summary results.

### The need for more indicators

Co-researchers recognised the need to support calculation of additional indicators (Supplemental Table S13), including more detail for public transport indicators (e.g. by mode); cycling-related indicators (a cycling working group to prototype was subsequently established); as well as air/noise pollution- and heat-related indicators (the latter are currently under development). The specific measures supported for analysis and reporting to-date are just a fraction of the possible indicators that would be relevant to planning healthy and sustainable cities (Giles-Corti et al., 2022). These focus on assessing ‘macro-scale’ accessibility: road connectivity, availability of public transport and proximity to destinations of interest, such as parks and healthy food outlets. However, micro-scale interventions such as paving, shade, signage and lighting are also important for supporting active lifestyles, and their evaluation is not currently supported. While our macro-scale indicators provide a robust overview of urban accessibility, we recognise they may overlook the nuances of local micro environments. Micro-scale factors can provide valuable insights into particular local conditions, but, on the other hand, they vary greatly across different geographic contexts, being more likely to be shaped by specific cultural values and political influences – such as the choice of asphalt paving (Sanders, 1984) – and, thus, are very challenging to standardise.

### Outputs and reporting

Co-researchers appreciated the commitment to support use of open access data to generate outputs in useful formats, for example, GeoPackage and CSV data files and PDF indicator and analysis reports. These features were considered important, with several users commending how this facilitated subsequent analysis (Supplemental Table S7). Co-researchers indicated that the produced reports were easy to understand and useful for sharing (Supplemental Table S11). One participant acknowledged the usefulness of the design but emphasised the need for customisation to effectively communicate with different audiences:

You don't need to be an expert, it's got the right amount of explanation … This could be used for urban planners or ... public health professionals… [but] My experience is that…the language or some things might ... [need to] be written to suit their perspective.

Another co-researcher pointed out the importance of providing additional contextual information for cities to guide interpretation of results (Supplemental Table S12).

in the current version you jump into the indicators result right away… maybe it would be nice to have some context information about the city, like … the population size… what forms of mass transit systems they have, if they have a light rail … some context information that allows you to understand or interpret or contextualize the results from the indicators … a picture of the city before jumping into the results.

It was also suggested that reports ‘*could be* [very useful] *for city councils to plan their urban master plans’;* however, there needs to be flexibility to also report on ‘*neighbourhoods, for districts, for municipalities*’.

Responding to this feedback, we implemented user-configurable fields for providing context on urban form, demographics, inequities, natural disasters and overall summaries interpreting the meaning of findings for each study region in generated reports. We also implemented the capacity to undertake distinct analyses and reports for sub-regions of interest, as required.

### Guidance

One co-researcher commented that changes to the online software documentation website had made the toolkit easier to use, with usage screenshots being ‘*really useful’*. However, at the time of the workshops, it was agreed that more guidance was required (Supplemental Table S9). It was suggested, ‘*videos are going to help a lot of people*’, and that the software, ‘*should have simple to follow directions*; [because] *ideally this should be for a person who has some technical expertise but not a computer science expert*’. The co-researcher who made the latter comment, a physical activity and environment researcher, provided themselves as a user example: ‘*I can learn, but I’m not an expert at managing technical software’*. This was echoed by another co-researcher:

I’m not necessarily tech savvy; I’m not an expert, but I’m also not a beginner, and I had a lot of difficulty. I tried to run the code or the scripts when the GitHub page was the only resource for it. This new page was not existing yet and now with the video that you made available, I think that's going to be very, very helpful.

Comments like these emphasised the importance of making the software easier to use and improving documentation, making this accessible through multiple mediums. The need for more visual modes of communication was emphasised:

a more visual way of understanding the steps, so that people can just see that…, ‘OK, I understand the process, I don't know what happens behind the scene[s], but visually I understand the steps’.

There were multiple requests for short videos, that should be ‘*scripted … pitched at right level, broken into steps*’. It was commented that the advantage of producing short, topic-specific videos was that these could be curated as a playlist, and as software is updated, segments could be updated. Additionally, it was considered that short videos would be easier to translate into other languages. Co-researchers recommended providing a summary/motivation at the start of videos to introduce what each video was going to cover. It was also noted that ‘*Some users will need a 5-minute video, others more detailed advice*’. Another advantage of instructional videos was that these could be included in the web page instructions. Additional specific requests were made for guidance on a protocol or processes for public communication of results, and more examples for incorporating specific data.

We produced and interspersed a series of short videos amongst expanded guidance in our software instructional website that introduces the software, its purpose and usage (https://healthysustainablecities.github.io).

## Discussion

### Developing an open science urban indicator toolkit through action research

We engaged subject matter experts from diverse urban contexts globally as co-researchers to develop an open science urban indicator toolkit for meeting their urban indicator calculating and reporting needs. Through this study we gained a better understanding of the users of an open science toolkit for urban policy and spatial indicators, their requirements and use cases. By actively soliciting and iteratively addressing feedback, we enhanced the toolkit’s accessibility and functionality. The resulting software and accompanying documentation are further described in Supplementary Material and at https://healthysustainablecities.github.io.

**Figure 2.**
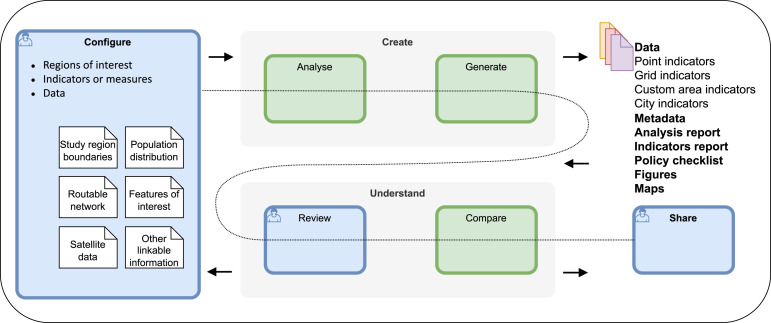
Four-step workflow for analysis and reporting of spatial indicators for healthy and sustainable cities.

The four-step workflow we developed through the course of this study is presented in Figure 2. This diagram illustrates how users are guided to retrieve and document required datasets in standard spatial formats through a study configuration file that is then used to perform urban indicator analyses and generate resources. Results (in the form of maps, reports and spatial data) are to be reviewed in conjunction with reporting on analysis assumptions and user-supplied context summaries. Analyses are iteratively revised to ensure valid representations of local context prior to publication and sharing as open data.

While the Observatory executive desired tools to support participation in the 1000 City Challenge, members of prospective city teams who would participate in the Challenge had their own goals: connecting local government planners and policy makers with bespoke indicators for their municipality and neighbourhoods; linkage of built environment exposures with population survey health data; before and after impact analyses for precinct redevelopments; and longitudinal monitoring to inform municipal urban master plans. Use cases such as these informed a list of software feature requests and requirements that were prioritised and implemented, or will be addressed in future updates through ongoing software development. Our work to identify and meet these goals is anticipated to lead to broader usage of the toolkit beyond participation in the 1000 Cities Challenge.

The toolkit was designed to support replicability and reproducibility in urban open science, ideals which remain an ongoing challenge in our field (Wilson et al., 2021; Wolf, 2023). To make our open-source software accessible for a broad audience of urban practitioners, we provided multiple modes of usage underpinned by a stable, curated computational environment for advanced spatial and network analyses and database management supported through our status as a Docker-sponsored Open-Source Software project. The recording of analysis provenance information through log files and detailed, accessible analysis reports provides transparency around the input data, assumptions and workflow and enhances reproducibility. The output of documented data in structured and open formats including GeoPackage and CSV files allows for interoperability with other workflows. This is further enhanced by provision of geospatial and ISO standard metadata in XML and YML formats. The additional provision of guided training materials means that the software, and its development process, also serves a pedagogical role.

Software development and maintenance is an ongoing process that must be approached incrementally (Farley, 2022). Enhancements to be implemented and bugs to be addressed have been progressively logged and resolved in our GitHub repository. Detailed instructional materials across multiple mediums have been provided. As the software toolkit is applied across diverse settings with variable data availability, formats and quality, it is anticipated that additional requirements will be identified for implementation in future software versions.

While our software has been developed as an open-source software project, most of our co-researchers were unable, or lacked confidence or desire, to engage deeply with the GitHub platform itself. This was not surprising given co-researchers identified primarily as applied urban policy and health researchers; only a subset reported having skills aligned with scientific computing. Most feedback received was through e-mails and online meetings – scheduled feedback workshops, training webinars, monthly executive and working group meetings and ad hoc meetings to discuss specific needs or issues as they arose. Due to the dispersion of our co-researcher cohort across multiple time zones, a flexible approach to online communication was essential.

There were only 20 formal co-researchers in the study. Observatory executive members (8/14 not completing the online survey) may not have felt the need or importance of engaging given their privileged opportunity to provide direct feedback via monthly meetings and e-mail. Co-researchers recruited as early adopters in the 1000 City Challenge (from Brazil, Chile, China, England, Mexico and Spain) or through direct expressions of interest to the lead author were enthusiastic contributors. However, this highly educated cohort was self-selected by interest in either using the software themselves or the outputs that would be generated. Co-researchers were not openly recruited due to concerns that engagement with a larger cohort would not be feasible. In the context of an action research study, the smaller cohort size allowed deeper engagement with 1000 Cities Challenge stakeholders than would have otherwise been possible. This is important, according to Stringer and Ortiz Aragón (2021), since effective action research is founded on quality relationships, stakeholder inclusion and honouring stakeholder perspectives. It would have been challenging to reach this level of inclusivity or achieve these three core principles with a much larger cohort of participants. However, there would be benefits from future research engaging to understand the needs of broader communities of individuals who share an interest in advocacy using urban indicators regardless of academic background, for example, for grass roots advocacy of interventions to address urban inequities.

The fusion of action research and software development may seem unusual, but they are conceptually linked as pragmatic methods for achieving progress through iterative action. Agile software development cycles involving continuous development, testing, deployment and review (Farley, 2022) align well with cycles of action, evaluation and reflection in action research (Stringer and Aragón, 2021). Solving problems through deep engagement and iterative, incremental actions guided by cycles of feedback are the common threads (Farley, 2022; Stringer and Aragón, 2021). However, ‘full participatory involvement’ (Stringer and Aragón, 2021) may be impossible to achieve in software engineering research. Applied co-researchers can only participate so much given skewed expertise and commitments towards their problem domain, and in most cases in this study, not to software architecture and programming. As such, for the research software engineer leading an action research study, there is a dual responsibility: acting as a catalyst for a group of stakeholders to realise solutions to their problems, but then also, acting as a translator to implement those solutions in code on the stakeholders’ behalf, due to their time and/or capability constraints.

### Contextualisation of policy and spatial indicators

Including both policy and spatial indicators in our software enables evaluation of whether, where and how stated policy objectives are being achieved in practice (Lowe et al., 2022). However, there is a fundamental difference between spatial and policy indicators. The former are regarded as concrete, objectively measurable descriptions of the built environment based on standardised secondary and open geodata. Policy indicators are qualitative, requiring detailed review of current policy literature at a point in time, and awareness of the ephemeral nature of policy (Lowe et al., 2022). Yet, policy and spatial indicators both involve interpretations of data- or prose-representations of places at particular timepoints and may require additional contextualisation to communicate their practical implications. With such a caveat, the policy and spatial indicator reports can serve as useful tools to evaluate delivery of both infrastructure and its enabling policy environment. Following co-researcher feedback, users can configure nuanced summaries to guide report reader’s understanding of what results mean for specific urban contexts as required. This is important as local researchers will be best placed to not only contextualise their findings but also prepare them using language and terms that engage and empower local policy makers with evidence to enact positive change (Choi et al., 2005). This approach of contextualising differences is in contrast to city-ranking tables, an approach that most co-researchers considered unhelpful for informing city planning policy and practice, and which we consciously avoided implementing.

### Identifying and addressing open data limitations

The limitations of open data (Kang et al., 2019) – which, like all data, can vary in quality and availability spatially and temporally – were acknowledged by participants. We implemented methods for configuring use of alternative open data for various urban features to meet this need for flexibility. Methods for configuring alternative pedestrian-network data than OpenStreetMap remain future work. Notwithstanding the need for flexibility, the lack of alternative official data is what drives interest in using OpenStreetMap. For many places in the world, it serves as the best or only available geospatial data on the built environment (Solís and Zeballos, 2023).

Global volunteer mapping communities and organisations such as the Humanitarian OpenStreetMap Team and YouthMappers coordinate mapping and validation efforts particularly in areas with poor data coverage and urgent need for accurate geospatial information, ideally with involvement of local communities (Herfort et al., 2021). Our collaboration has been actively seeking engagement with such organisations, whose international networks could participate with local teams for validation and mapping, while benefitting from the features offered by our toolkit for their own research, planning and advocacy purposes.

Validation of input data is an important area that co-researchers requested guidance on (Supplemental Table S10). Currently, users can generate an analysis report to review the assumptions and implications of their region configurations, along with the distribution of features in their study regions. Guidance is provided for accessing the generated data for further review within a desktop GIS software for manual evaluation and comparison with reference data. For the prototype 25 cities study, analyses were conducted by a central team in consultation with local experts who were provided with preliminary validation reports that described the methods used to configure study region boundaries and represent features of interest, and visualise the implications of this in maps (phase 1 validation), and through checks with reference data and imagery (phase 2 validation) (Boeing et al., 2022; Liu et al., 2021). At the time of the workshops, phase 1 validation had not yet been re-implemented in the updated software. However, subsequently updated methods for users to generate separate analysis reports for both spatial and policy indicators were incorporated along with other guidance and tools to support validation (Supplemental Table S10). Further support for user validation remains an important aspect for future development.

### Knowledge translation for local audiences using a shared methodology, globally

The will to standardize indicators across the globe was treated with necessary care. Complex and multifactorial issues such as the relationship between urban design, transport and population activity and health indicators are not always objectively measurable. Encouraging users to carefully review and contextualise the reporting of policy- and spatial indicator results mitigates the risk of oversimplifying complex social phenomena. Further, engaging the administrative sphere with scientific evidence is challenging in many cultures. Empowering local experts and communities to act within an established global network can help to lower these barriers, using a shared toolkit that can be configured to produce a range of contextualised outputs that support knowledge translation for diverse audiences (Jull et al., 2017). Finally, we wanted to push back against neo-colonial or ‘parachute’ science (Helm et al., 2023), where centralised analysis and generation of urban planning and public health knowledge is undertaken unilaterally by teams, particularly in developed Western countries, and imposed as uncritical truths onto other regions of the world. This toolkit was developed in the spirit of open science, through engagement with co-researchers from diverse global contexts, to empower local teams to represent and share their own urban realities both locally and globally.

## Conclusion

Using action research principles to guide collaboration, an integrated open science urban indicator toolkit was developed to support participation in the Observatory’s 1000 Cities Challenge. This open-source software is designed to meet the needs of researchers, planners, policy makers and community advocates in diverse settings for planning, calculating and disseminating policy and spatial urban indicators. In doing this, it delivers multiple streams of evidence required by multiple actors to collectively influence policy agendas and enact policy change, and thereby generate momentum for an urgently needed transition towards healthier, more equitable and sustainable cities.

More work is required: new spatial indicators to measure the delivery of policies related to climate change risk and resilience; additional guidance, tools and reporting to support user validation; and greater flexibility for within-city comparisons to engage local stakeholders. The delivery of ‘software’ run on a local computer itself is a paradigm that could be questioned with advances in artificial intelligence and ubiquitous cloud computing. However, the former is still nascent and lacking transparency, while the latter assumes access to server resources which require on-going funding.

The main objectives of this study were to develop an integrated open-source tool that delivers the multiple streams of evidence required by multiple actors to collectively influence policy agendas, and supports participation of local teams in cities globally to benchmark and monitor urban policies designed to create healthy and sustainable cities. Through this action research project, we have met our aim to deliver working software that meets our current urban planning and health stakeholder needs. This research was conducted in the spirit of recommendations for broader adoption of the principles of open science (UNESCO, 2021), that emphasise inclusivity, rigour, transparency and respect. We have consciously designed our Global Healthy and Sustainable City Indicators tool with these ideals in mind to support urban policy and public health practitioners to calculate, analyse and report for local stakeholders on policy and spatial indicators of urban design and transport features using a reproducible open-source software workflow. The creation of accessible, detailed, well-documented data and method reports tailored for local audiences promotes a broad range of community stakeholders’ engagement with the challenges and solutions for creating equitable, healthy and sustainable cities.

## Supplemental Material

Supplemental Material - Global Healthy and Sustainable City Indicators: Collaborative development of an open science toolkit for calculating and reporting on urban indicators internationally Supplemental Material for Global Healthy and Sustainable City Indicators: Collaborative development of an open science toolkit for calculating and reporting on urban indicators internationally by Carl Higgs, Melanie Lowe, Billie Giles-Corti, Geoff Boeing, Xavier Delclòs-Alió, Anna Puig-Ribera, Deepti Adlakha, Shiqin Liu, Julio Borello Vargas, Marianela Castillo-Riquelme, Afshin Jafari, Javier Molina-García, Vuokko Heikinheimo, Ana Queralt, Ester Cerin, Eugen Resendiz, Dhirendra Singh, Sebastian Rodriguez, Esra Suel, Marc Domínguez-Mallafre, Yang Ye and Amanda Alderton in Environment and Planning B: Urban Analytics and City Science.

## Data Availability

The Global Healthy and Sustainable City Indicators tool is available from https://healthysustainablecities.github.io (Higgs et al., 2023a). The survey questionnaire and summary of results are included in Supplementary Material. De-identified survey data are available at https://doi.org/10.25439/rmt.24494563. Code used for analysis is available at https://doi.org/10.25439/rmt.24494647.

## ORCID iDs

Carl Higgs https://orcid.org/0000-0002-5903-9095

Melanie Lowe https://orcid.org/0000-0002-9703-1895

Billie Giles-Corti https://orcid.org/0000-0003-0102-0225

Geoff Boeing https://orcid.org/0000-0003-1851-6411

Xavier Delclòs-Alió https://orcid.org/0000-0002-7206-2310

Anna Puig-Ribera https://orcid.org/0000-0003-2910-1924

Deepti Adlakha https://orcid.org/0000-0002-1720-6780

Shiqin Liu https://orcid.org/0000-0001-9605-3251

Júlio Celso Borello Vargas https://orcid.org/0000-0001-8321-5362

Marianela Castillo-Riquelme https://orcid.org/0000-0002-6933-4602

Afshin Jafari https://orcid.org/0000-0001-5153-2179

Javier Molina-García https://orcid.org/0000-0001-6713-5936

Vuokko Heikinheimo https://orcid.org/0000-0001-5119-0957

Ana Queralt https://orcid.org/0000-0003-4933-0003

Ester Cerin https://orcid.org/0000-0002-7599-165X

Eugen Resendiz https://orcid.org/0000-0002-4500-879X

Dhirendra Singh https://orcid.org/0000-0001-8369-8878

Sebastian Rodriguez https://orcid.org/0000-0002-0514-9221

Esra Suel https://orcid.org/0000-0001-9246-3966

Marc Domínguez-Mallafré https://orcid.org/0000-0003-0893-170X

Yang Ye https://orcid.org/0000-0003-2642-0436

Amanda Alderton https://orcid.org/0000-0002-4598-2381
