# Supplementary material for: Global Healthy and Sustainable City Indicators: Collaborative development of an open science toolkit for calculating and reporting on urban indicators internationally
Source: Environ Plan B Urban Anal City Sci. 2024 Dec 2;52(5):1252–70. doi: 10.1177/23998083241292102 (PMC13035218; doi:10.1177/23998083241292102)
Supplement: Supplemental Material - Global Healthy and Sustainable City Indicators: Collaborative development of an open science toolkit for calculating and reporting on urban indicators internationally [file sj-pdf-1-epb-10.1177_23998083241292102.pdf]

Collaborative development of an open science urban indicator toolkit for assessing healthy and sustainable cities internationally: Supplementary Material

Global Healthy and Sustainable City Indicators tool

The software designed through this study can be configured to calculate and report on policy and spatial indicators for healthy and sustainable cities in diverse contexts globally. The core set of spatial indicators are calculated for point locations, a small area grid (e.g. 100m), and overall city estimates. Optionally, indicators can also be calculated for custom areas, like administrative boundaries or specific neighbourhoods of interest. In addition CSV files containing indicators for area summaries and the overall city are also generated, omitting geometry. Metadata and data dictionaries are generated to accompany the data, along with reports in multiple languages.

The default core set of spatial urban indicators calculated includes:

- Urban area in square kilometres
- Population density (persons per square kilometre)
- Street connectivity (intersections per square kilometre)
- Access to destinations within 500 meters:
  - a supermarket
  - a convenience store
  - a public transport stop (any; or optionally, regularly serviced)
  - a public open space (e.g. park or square; any, or larger than 1.5 hectares)
- A score for access to a range of daily living amenities
- A walkability index

The tool can also be used to summarise and visualise policy indicators data collected using the [1000 Cities challenge policy checklist tool].

The resulting city-specific resources can be used to provide evidence to support policy makers and planners to strengthen urban policy, target interventions within cities, compare performance across cities, and when measured across time can be used to monitor progress towards achieving urban design goals for reducing inequities. Moreover, they provide a rich source of data for those advocating for disadvantaged and vulnerable community populations.

Generated outputs include:

- Summary of configuration parameters used for analysis (.yml file)
- Processing log detailing the analyses undertaken (.txt file)
- Geopackage of indicator results and spatial features including points and areas of interest and pedestrian network (.gpkg)
- CSV files for indicator results (.csv)
- Data dictionaries (.csv and .xlsx files)
- ISO19115 metadata (.xml and .yml files)
- Analysis report (pdf)
- Policy and spatial indicator report, optionally in multiple languages (.pdf)
- Figures and maps, optionally in multiple languages (.jpg)

The software is designed to be used by local experts as part of multi-disciplinary teams participating in the 1000 Cities Challenge; but anyone (e.g. students, enthusiasts) can use the open source software.

Action research-informed software development

We developed an action research framework to support development of urban analysis software that would be accessible and useful for our stakeholders. Action research is a qualitative participatory research methodology concerned with delivering tangible outcomes that address real world problems (Stringer and Aragón, 2021). It aligns with the values and principles of the Agile manifesto for software development (Beck et al., 2001), a method for iterative delivery of functional software that meets the needs of users through reflection, adjustment and evaluation. Action research and Agile principles both emphasise the role of respectful collaboration to better understand a community's requirements. We created the conceptual diagram in **Error! Reference source not found.** to illustrate how building shared knowledge of a project's goals facilitates their realisation through iterative and incremental progress. Research participant 'co-researchers' led the development of working software through iterative cycles of observing, thinking, and acting, resulting in progressive refinement of prototypes to meet stakeholders' urban indicator reporting needs.

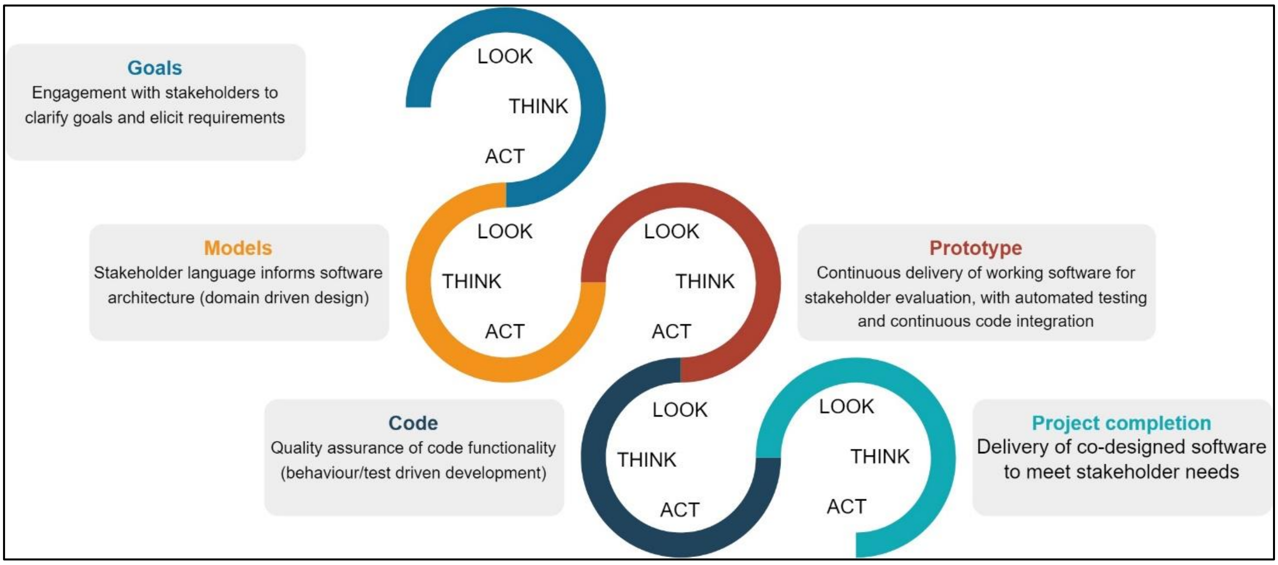

Figure S1. Iterative process of improvement of working software that meets stakeholder goals.

To understand our diverse participants' backgrounds and needs, we designed an online survey to capture their professional and demographic attributes and requirements (Table S1). Invitations to participate in the survey and follow-up online focus groups were shared via e-mail. Completion of the baseline survey or participation in recorded workshop sessions indicated informed consent. Participants were also invited to engage directly in software development through online discussions, lodge 'issues' to request new features or report bugs via the public GitHub site (https://github.com/healthysustainablecities/global-indicators), or e-mail queries and initiate ad hoc meetings to facilitate their usage of the software. Through these mediums, participants provided on-going evaluation and feedback on project goals and their implementation.

Participants initially contacted in March 2023 (n=24) were invited to one-hour workshop feedback sessions, respectively focused on the process of using the software, and reporting requirements. The workshops were repeated across different time zones and conducted online via Microsoft Teams, video-recorded, and auto-transcribed. Following each of the workshops, video recordings were shared with participants along with summaries of the points raised via e-mail and on the GitHub software repository discussion board. Participants had the opportunity to further contribute or amend their recorded suggestions as required.

Software development

A website was established that explained how to download, install and run the prototype software to calculate and report on urban indicators. Addressing prior feedback, the website aimed to avoid jargon and explain usage in clear terms, so as not to alienate users unfamiliar with using code to run analyses or develop open-source software. This was important, as *a priori* the software aimed to provide an accessible, rigorous and reproducible means for users to report on urban policy and spatial indicators, while minimising technical hurdles. Along with the software, this website was updated throughout the course of the study following participant feedback on their experiences and needs. Data collected from engagements between the researchers and participant co-researchers resulted in agreed actions that guided the software development project according to the model in Figure S1.

As detailed in the main article, following participant feedback, four modes of usage were implemented for analysing configured urban study regions and generating reports and resources using the installed software: via a web browser, through a user-friendly app (Figure S2); as command-line prompts ('configure', 'analysis', 'generate', and 'compare'); as a Python module for advanced flexibility; or within the provided Jupyter Lab app (https://jupyter.org).

Figure S2. The Global Healthy and Sustainable City Indicators application, running locally in a web browser offers a simple graphical user interface for managing study region configurations, running analyses, generating resources, comparing scenarios or study regions as well as querying and exporting completed policy checklists

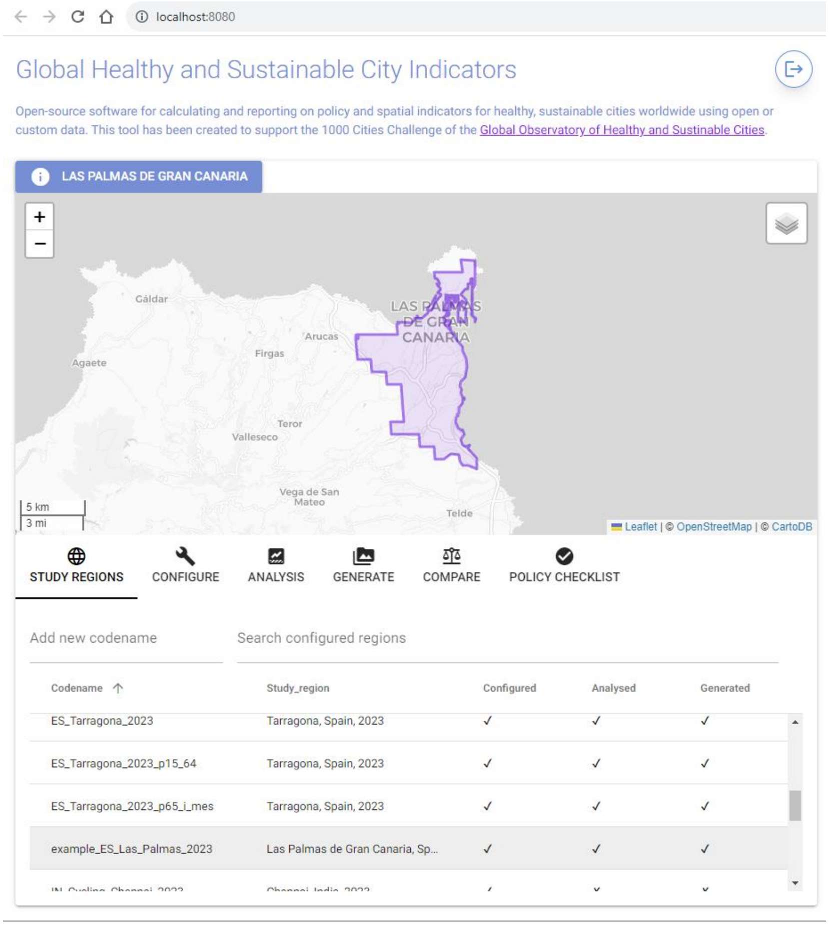

Survey analysis methods

The Google Forms survey was used to characterise the cohort and provide quantitative summaries of experience and requirements, while understanding how these relate to specific user contexts. Responses were exported as an Excel file. A de-identified set of responses was used for analysis, redacting fields containing participant contact information or identifying specific universities. Sensitivity analyses were conducted to evaluate the results both for the full cohort and omitting the Observatory executive members (Table S2 and Supplementary Material). Descriptive statistical analyses of survey data were conducted using the Anaconda distribution of Python (3.8.16) within a Jupyter notebook with the Jupyter Lab (3.5.3) interface using the Pandas (2.0.1), Scipy (1.10.1), Matplotlib (3.7.1) and openpyxl (3.0.7) libraries. Rank correlations were calculated using Kendall's Tau (τ), a non-parametric estimate for strength of association that avoids distributional assumptions.

Table S1. Participant survey items

#	Format

#		Format
1	Professional role	Text
2	Years of experience in this profession	Integer
3	In what city and country do you work or study (for example, "Melbourne, Australia")	Text
4	Languages used in professional capacity	Text
5	Please rate your experience in the following areas from 1 (least experienced) to 7 (most experienced)	7-point Likert
	Creating or analysing policy	-
	Spatial or network analysis	-
	Using open data	-
	Publishing open data	-
	Using research evidence to inform policy development	-
	Stakeholder engagement and lobbying	-
6	Who do you envisage will use this tool?	Text
7	What would you ideally like the tool to produce?	Text
8	How important are the following features for you in a tool? Please rate from 1 (not important) to 7 (very important).	7-point Likert
	Helping to collate city planning policy data/references	-
	Helping to collate city planning spatial data	-
	Measuring policy indicators	-
	Measuring spatial indicators	-
	Adding new indicators	-
	Validating indicators	-
	Within city comparisons of policy and spatial indicators	-
	Between city comparisons of policy and spatial indicators	-
	Ability to be customised for diverse contexts	-
	Integrated project planning	-
	Generating spatial indicator maps	-
	Generating reports	-
	Generating graphs	-
	Generating data	-
	Generating metadata and other documentation	-
	Reporting in different languages	-
	User interface in different languages	-
9	Please list any other important features for you if these are not listed above	Text
10	Will you, yourself, use this tool?	Multiple choice (single answer)
	Yes	-
	No	Skip to Q11
	Maybe	-
10a	How would you like to run this software? Please indicate your preference by ranking the following options least likely (1) to most likely (6) to use	Rank order - conditional on Yes/Maybe in Q10
	As a 'point and click' desktop application	-
	As a cloud application (e.g. a tool on a web site)	-
	As an API (ie. a program with functions that can be used by other software)	-
	As a software module/library/package	-
	As a methodology (ie. using the general process, but not necessarily as software)	-
	As code (e.g. a Jupyter notebook)	-
10b	Which, if any, programming language (s) would you like to use this software with?	Text - conditional on Yes/Maybe in Q10
11	Will someone else you know use this tool?	Multiple choice (single answer)
	Yes	-
	No	Skip to Q12
	Maybe	-

#			Format
11a	If appropriate, please invite other potential users of the tool to participate in this study. Please supply e-mail addresses and we will invite them to participate advising that they have been nominated as a potential user of the tool.		Text - conditional on Yes/Maybe in Q11
12	How do you envisage the tool being used?		Text
13	Do you have any further comments about the proposed tool?		Text
14	What is your age group?		Multiple choice (single answer)
		or younger	-
		20-29	-
		30-39	-
		40-49	-
		50-59	-
		or older	-
15	How do you describe your gender? (Gender refers to current gender, which may be different to sex recorded at birth and may be different to what is indicated on legal documents)		Multiple choice (single answer)
		Man or male	-
		Woman or female	-
		Non-binary or other term preferred	-
		Prefer not to say	-
16	What is your highest formal educational qualification?		Text

Table S2. Grouped summaries of participant demographic and continuously valued responses as counts and distribution summaries, with results for full cohort and sensitivity analysis excluding executive group members (n=6)

		**Full cohort**		**Excluding executive group members (n=6)**	
		**n, or #**	**%**	**n, or #**	**%**
	Number of participants (N)	17	100.0	11	100.0
Primary professional role (N)	Researcher	7	41.2	4	36.4
	Professor	4	23.5	3	27.3
	Research fellow	2	11.8	1	9.1
	Consultant	1	5.9	1	9.1
	Technician	1	5.9	1	9.1
	Lecturer	1	5.9	1	9.1
	Doctoral student	1	5.9	0	0.0
Years of experience in this profession (distribution summary #; no missing values)	mean	10	-	11	-
	std	6	-	6	-
	min	1	-	1	-
	25%	6	-	6	-
	50%	10	-	13	-
	75%	15	-	17	-
	max	20	-	20	-
In what city and country do you work or study? (N)	Spain (Valencia, Vila-seca, Vic)	5	29.4	5	45.5
	Australia (Melbourne)	3	17.6	1	9.1
	México (Cuernavaca, Mexico City)	2	11.8	1	9.1
	United States of America (Los Angeles, Minneapolis)	2	11.8	0	0.0
	United Kingdom (London)	1	5.9	1	9.1
	Chile (Santiago)	1	5.9	1	9.1
	China (Harbin)	1	5.9	1	9.1
	Finland (Helsinki)	1	5.9	0	0.0
	Brazil (Porto Alegre)	1	5.9	1	9.1
Languages used in professional capacity (N)	English	17	100.0	11	100.0
	Spanish	9	52.9	8	72.7
	Catalan	5	29.4	5	45.5
	Chinese	1	5.9	1	9.1
	Finnish	1	5.9	0	0.0
	Italian	1	5.9	1	9.1
	Portuguese	1	5.9	1	9.1
What is your age group? (N)	20-29	2	11.8	1	9.1
	30-39	9	52.9	5	45.5
	40-49	4	23.5	3	27.3
	50-59	2	11.8	2	18.2
How do you describe your gender? (N)	Man or male	8	47.1	6	54.5
	Woman or female	8	47.1	4	36.4
	Not answered	1	5.9	1	9.1
Highest education level attained (N)	PhD	14	82.4	10	90.9
	Masters	2	11.8	0	0.0
	Undergraduate	1	5.9	1	9.1
Will you, yourself, use this tool? (N)	Yes	16	94.1	10	90.9
	Maybe	1	5.9	1	9.1
Which, if any, programming language(s) would you like to use this software with? (N)	Python	7	41.2	3	27.3
	R	4	23.5	3	27.3
	Javascript	1	5.9	1	9.1
	Other responses (not answered or spoken language)	9	52.9	7	63.6

Prose summary of participant survey results

Demographic attributes

A total of 17 participants completed the online enrolment survey (Table S1), six of whom (35%) were executive members of the Observatory. Summaries of demographic and continuously valued responses are provided in Table S2. Most participants (13/17; 76%) had spent 6 or more years in their profession, and some reported having multiple roles. Experience ranged from 1 to 20 years, with a median of 10 years and interquartile range from 6 to 15 years. While participant ages ranged between 20 and 60, most participants (13/17; 76%) were aged in their 30s (9/17; 53%) or 40s (4/17; 24%). Approximately half the cohort identified as female (8/16; 50% of responders). The cohort was highly educated with all having completed tertiary education and most (14/17; 82%) having a PhD degree. Excluding executive members resulted in a small upwards shift in the remaining 11 study participants reported experience (median of 13 years; interquartile range, IQR, 6 to 17 years), and fewer participants identifying as female (4/11; 36%).

Participants' primary location of work was situated in 13 cities across 9 countries, spanning the continents of Europe (n=7; Spain, Finland and United Kingdom), North America (n=4; Mexico and United States of America), Oceania (n=3, Australia), South America (n=2; Brazil and Chile) and Asia (n=1, China). In addition to English, which all participants used professionally, more than half (n=9) used Spanish, five used Catalan, while individuals also reported using Chinese, Finnish, Italian and Portuguese professionally.

Urban analysis and reporting experience

Survey participants were asked to rate their experience undertaking a series of tasks related to creating and reporting on policy and spatial urban indicators for healthy and sustainable cities from 1 (least experienced) to 7 (most experienced); counts and correlations between tasks are summarised in Figure S2. Responses revealed the diversity of participants' professional experience. Approximately half (9/17; 53%) rated their '*Spatial or network analysis*' experience in the top two tiers, with only two (12%) in the lowest two tiers. In contrast, experience ratings for '*Creating or analysing policy*' were polarised, with seven participants (41%) reporting in each of the two highest and lowest experience tiers. Approximately half of participants indicated confidence in using open data (8/17 rating in the highest two tiers; 47%), yet few (3/17; 18%) were similarly confident in publishing open data. While fewer non-executive group members rated having experience in the highest two tiers of creating/analysing policy and analysis spatial/network data (4/11 each, 36%; compared with 41% and 53% for the full cohort), associations observed between experience categories remained broadly similar.

To characterise associated patterns of cognate tasks, a matrix of rank correlations was calculated using Kendall's Tau (τ). Confidence in publishing open data had a moderately strong negative correlation with

confidence in creating or analysing policy (Kendall's τ coefficient of -0.27), but moderate positive correlation with using open data (τ=0.32) and conducting spatial or network analysis (τ=0.32). Using open data had a strong positive correlation with spatial/network analysis (τ=0.88), but a weak negative correlation with creating or analysing policy (τ=-0.18). Policies were listed in Figure S2 in ascending order according to their correlation with 'Creating or analysing policy' (the most polarising task for the full cohort). Exclusion of the executive group resulted in a downward shift in reported experience across all tasks, and shift in the ordered list of correlations (Figure S2).

a) Full cohort (n=17)

Task (*ordered by 'Creating or analysing policy' rank correlation*)	Experience rating counts: 1 (*Least*)	2	3	4	5	6	7 (*Most*)	Kendall's rank correlation (τ): Publishing open data	Using open data	Spatial or network analysis	Stakeholder engagement and lobbying	Using research evidence to inform policy development	Creating or analysing policy
Publishing open data	2	2	3	4	3	1	2	1.00	0.32	0.32	0.09	-0.01	-0.27
Using open data	0	2	1	4	2	4	4	0.32	1.00	0.88	-0.09	-0.06	-0.18
Spatial or network analysis	0	2	2	4	0	4	5	0.32	0.88	1.00	-0.04	0.07	-0.04
Stakeholder engagement and lobbying	2	5	4	1	1	3	1	0.09	-0.09	-0.04	1.00	0.27	0.24
Using research evidence to inform policy development	1	1	1	4	5	3	2	-0.01	-0.06	0.07	0.27	1.00	0.24
Creating or analysing policy	5	2	1	1	1	6	1	-0.27	-0.18	-0.04	0.24	0.24	1.00

Key

Negative

Neutral

Positive

b) Sensitivity analysis excluding executive group (n=11)

Task (*ordered by 'Creating or analysing policy' rank correlation*)	Experience rating counts: 1 (*Least*)	2	3	4	5	6	7 (*Most*)	Kendall's rank correlation (τ): Using open data	Publishing open data	Spatial or network analysis	Using research evidence to inform policy development	Stakeholder engagement and lobbying	Creating or analysing policy
Using open data	0	2	0	4	2	2	1	1.00	0.26	0.79	-0.39	-0.19	-0.19
Publishing open data	2	1	3	3	2	0	0	0.26	1.00	0.30	0.11	0.35	-0.17
Spatial or network analysis	0	2	1	4	0	3	1	0.79	0.30	1.00	-0.24	0.00	0.00
Using research evidence to inform policy development	1	1	0	2	4	2	1	-0.39	0.11	-0.24	1.00	0.65	0.21
Stakeholder engagement and lobbying	2	3	2	0	1	2	1	-0.19	0.35	0.00	0.65	1.00	0.31
Creating or analysing policy	3	2	1	0	1	3	1	-0.19	-0.17	0.00	0.21	0.31	1.00

Figure S3. Summary counts and rank correlation matrix for reported experience of (a) the full participant cohort, and (b) excluding the executive group, across a series of tasks related to creating and reporting on policy and spatial urban indicators for healthy and sustainable cities, presented in ascending order according to correlations for the task 'Creating or analysing policy' (most polarised in terms of reported experience).

Audience for an open science urban indicators toolkit

It was envisaged that an open science toolkit for policy and spatial urban indicators would be used by urban health researchers, planners, policy makers, architects, data scientists and community advocates, in addition to municipal and supra-municipal governments, public health agencies, non-governmental organisations, and consultancies (see Qualitative survey responses). Nearly all participants (16/17; 94%) indicated that they themselves would use the proposed tool (Table S2), with the remaining participant indicating '*Maybe*' (1/17; 6%).

What should an open science urban indicators toolkit produce?

When asked what the tool should produce, there was a correspondingly broad set of expectations, from tangible outputs such as documented data, maps, and reports to more abstract concepts such as indicators, insights, comparisons and a streamlined workflow (see Qualitative survey responses). While many participants indicated a desire for reports and visualisations that are easy to generate and interpret, one emphasised the value of "*…new data/information that can be 'curated' and published according to the local dissemination needs*". This diversity of needs reflected the diverse backgrounds and experiences of respondents, and could indicate the heterogenous expectations and needs of an anticipated broad audience both in terms of professional background, but also interests, motivations and geographical context.

What features are most and least important?

Participants were asked to rate the importance of tool features on a scale from 1 to 7 (Table S2). The features rated highest were '*generating maps*', '*measuring spatial indicators*', and generating each of *'…data', '… reports'* and *'… graphs*'. Customisation for diverse contexts was also recognised as important, along with capacity for between and within-city comparisons and generating metadata and related documentation. Overall, non-executive members prioritised generating data above maps and measuring spatial indicators, reflecting intent to use calculated measures for local research and dissemination activities, beyond the 1000 Cities Challenge agenda.

Language-related features for UI-interaction and reporting were rated lower overall (Table S3); however, the stratified box-plots in Figure S3 demonstrate that users working with languages other than English valued these features' inclusion highly. The divergence was more pronounced with exclusion of the executive group (Figure S3b), whose core motivation was to design a tool that would be useful for users to measure cities in diverse global settings. These results suggest that language supporting features would be highly valued by many users and audiences using professional languages other than English, particularly for reporting in different languages.

Table S3. Mean, standard deviation (SD) and distribution summary (minimum, 25th, 50th and 75th percentiles and maximum) of feature importance ratings by the full participant cohort (n=17) for an open science urban indicators toolkit, ordered by mean rating.

Features	Mean	SD	Distribution summary				
			min	25%	50%	75%	max
Generating spatial indicator maps	6.5	0.9	4	7	7	7	7
Measuring spatial indicators	6.5	0.8	4	6	7	7	7
Generating data	6.5	1.2	3	7	7	7	7
Generating reports	6.5	1.5	1	7	7	7	7
Generating graphs	6.4	1.5	1	6	7	7	7
Ability to be customised for diverse contexts	6.2	1.3	3	6	7	7	7
Between city comparisons of policy and spatial indicators	6.2	0.9	4	6	6	7	7
Within city comparisons of policy and spatial indicators	6.2	1.0	4	6	6	7	7
Generating metadata and other documentation	6.1	1.2	3	6	6	7	7
Helping to collate-related city planning spatial data	6.1	0.9	4	6	6	7	7

Features	Mean	SD	Distribution summary				
			min	25%	50%	75%	max
Validating indicators	5.9	1.0	4	5	6	7	7
Adding new indicators	5.7	1.1	4	5	6	7	7
Measuring policy indicators	5.5	1.3	3	5	6	7	7
Helping to collate city planning-related policy data/references	5.5	1.4	3	4	5	7	7
Reporting in different languages	5.2	2.1	1	4	6	7	7
Integrated project planning	4.8	1.8	1	4	5	6	7
User interface in different languages	4.4	2.1	1	3	5	6	7

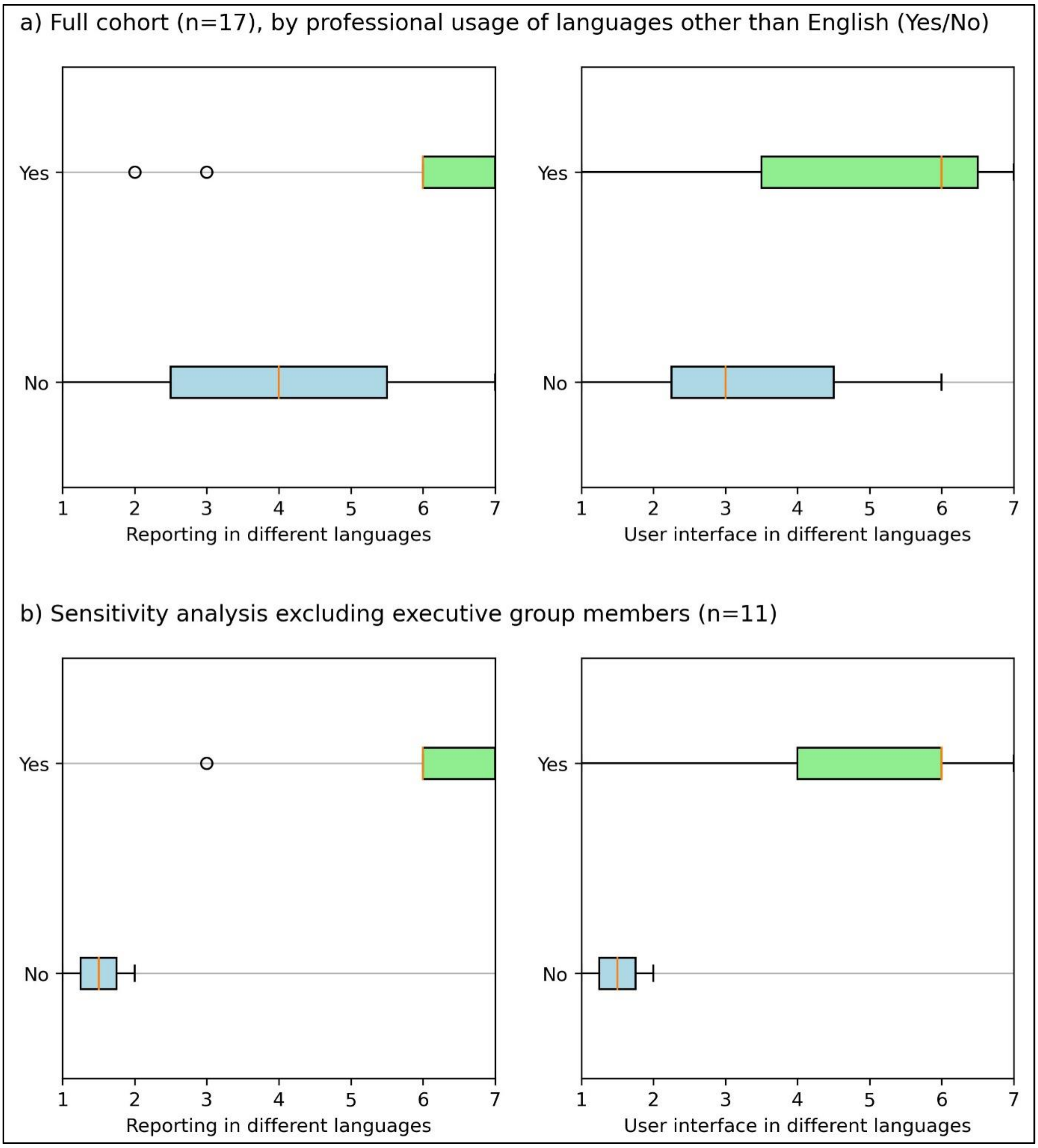

Figure S4. Disaggregation of importance ratings for language related features by professional usage of languages other than English status (Yes/No) for (a) full cohort (n=17), and (b) excluding the executive group (n=11).

User preferences for running software

Non-executive members had a preference to use the tool as a desktop- or cloud application; executive group members tended to prefer usage as a software module or cloud application (Figure S4). Some users were interested in the overall methodology, however few participants were interested in the concept of an API (the implementation of which could support development of more elaborate interfaces). All usage modes received at least some preference scores of 5 or greater, indicating diversity in the preferred mode of usage amongst the various participants. Despite the relative low preference ranking for usage as code, approximately half of the cohort (8/17; 47%) indicated that they would like to use the software with some kind of programming language (Table S2. Grouped summaries of participant demographic and continuously valued responses as counts and distribution summaries, with results for full cohort and sensitivity analysis excluding executive group members (n=6)), with some users specifying several candidates; primarily, Python (7/17; 41%) or R (4/17; 23.5%).

A minority of participants (4/17; 24%) offered additional comments (see Qualitative survey responses). Beyond positive feedback for the software and the participatory study, additional features were requested: automated downloading of population and OpenStreetMap data, and capacity for a customisable range of spatial scales for calculation of indicators ("*census tract, neighborhood, district, municipality*").

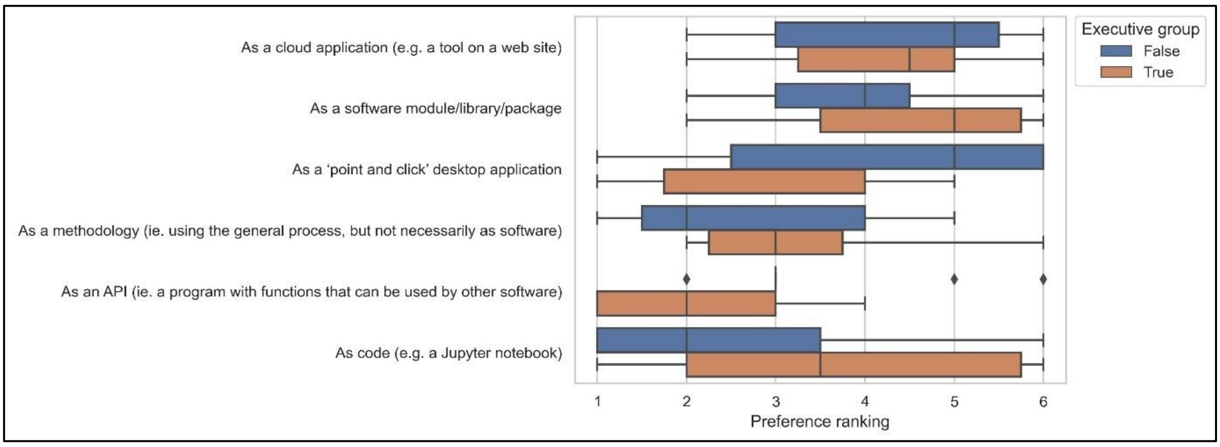

Figure S5. Box plots of participant ranking of preferred software usage mode, but executive group membership.

Qualitative survey responses

These qualitative responses are provided as direct quotes including provided punctuation and spelling.

Table S4. Survey participant responses to anticipated users for a proposed open science urban indicators toolkit

**Who do you envisage will use this tool?**
**Non-executive group responses (10/11)**
Researchers, Government agencies
Researchers, urban planners, policy makers, architects, etc.
Mainly researchers but also city planners or technicians of diverse areas of interest.
researchers from academia and professionals from
Research team
Myself, for my research in urban mobility and accessibility, as well as a larger team of Spanish collaborators in this area
Municipalities, Supramunicipality organizations, Public Health Agencies, Urban Services.
researchers, government
Planners and policy makers, urban researchers
Academic research to inform policy, advocacy, publishing evidence, instruction. '],
**Executive group responses (6/6)**
Population health and urban planning enthusiasts, in academia, government or consultancies
Researchers, data analysts, consultants, city planners, advocacy NGO
Non-profit organizations, community advocates and research groups
City planners, academic researchers, policy analyst
Geospatial data scientists
citizen scientists, city planners, policymakers

Table S5. Outputs of the proposed open science urban indicators toolkit desired by responding survey participants (16/17)

**What would you ideally like the tool to produce?**
**Non-executive group (10/11)**
Comparable and actionable insights to highlight priority areas for policy and research
I would like the tool to produce reports on spatial indicators comparing different parts of the same city. Of course, these reports should include graphs and visual information.
I would like the tool to provide visual, attractive, updateable and editable reports, as well as detailed databases with indicators, figures and descriptive tables both for each city and comparing with other cities.
Summary reports and graphs for indicators
We would like the tool to produce not only city-level and hexagon-level maps, but also reports at other relevant geographies (e.g. neighborhoods, districts), or to choose what spatial unit to be used.
High resolution maps of the spatial indicators explored in the 25-city project, at the city level, the hexagon level, as well as at other at other relevant geographies (e.g. neighborhoods, districts)
To identify and map urban liveability indicators for specific urban areas. If the mapping is done before and after the implementation of a urban intervention, ths tool will be great for evaluating the health impact of such interventions.
a feasible comparison tool for global city
A streamlined workflow to create built-environment indicators for different cities, easy to understand maps and reports to disseminate the findings
I am more interested in the method, protocols, standards, guidelines and codes for generating standardized indicators, and less in the 'ready-made' outputs such as maps and graphs. Therefore, I believe the software should fundamentally produce new data/information that can be 'curated' and published according to the local dissemination needs."
**Executive group (6/6)**
well documented data, reports, infographics, maps and plots in different file formats and languages to serve different purposes related to supporting evidence-based planning for healthy and sustainable cities
Easy to understand reports showing how a city is performing, and areas for improvement.
Downloadable /editable datasets, "easy" to interpret graphs
indicators to track and benchmark
Policy relevant, reliable insights about sustainable and healthy urban environment. Longitudinal analysis (reliable comparisons over time) would be super valuable.
descriptive evidence and useful reports

Table S6. Optional additional comments left by some (n=4) survey participants, by executive group membership status

**Other comments**
**Non-executive group**
It would be more easily to use if the tool downloads the data automatically (population and OpenStreetMap). The. yml files can be complicated to edit if you are not familiar with programming.
I think it's already a great tool! A couple of things that I'd like to add, if possible: possibility to change the spatial units at which indicators get calculated (census tract, neighborhood, district, municipality).
Executive group
I think the participatory process is important to ensure that users have ownership and a stake in the tool, and that their diverse needs are identified and hopefully met (at least, as far as possible). This will also create understanding amongst the group of stakeholders of the diversity of perspectives on the tool and what it could do and help guide its future development trajectory. I believe this is important research in itself.
Awesome tool, and great that it is developed further in a participatory manner :)

Consolidated feedback summaries from user workshops

Table S7. Software usability

Topic	Actions undertaken
Commitment to open access data (e.g. gpkg/csv), reports (e.g. PDF) and indicators (e.g. methods detail with references) is important; output with geopackages great, allows use in other software; gives flexibility; gets analysis moving forwards	positive feedback
Tools is simple to use for those with geospatial expertise, except for configuration	Simplified configuration and improved documentation; drafted approach for configuration in web app browser, to be implemented in future.
YAML Configuration is complicated and needs more guidance/examples	More guidance provided
Could split regions as separate city-specific configuration files, so these are less overwhelming	City-specific configuration
Currently the configuration contains examples for every region - is a bit confusing	Provided single exemplar
Show users how to modify configuration file with example, including copying and pasting sections	Tutorial website, notebook and video
Making tool so easy to use that applied users who are not technical specialists could use; under the hood may be complex, but important to not make it too complicated to use	'point and click' web app interface
Could have simple/advanced configuration or UI	Four modes of usage, for different users (app, command-line interface, python module, Jupyter notebook
Software usable as a Python library, e.g. could be run in a Jupyter notebook	Can use as python module; Jupyter notebook with usage examples provided
How easy can we go while being meaningful/useful?	Four modes of usage each providing documented outputs including metadata and provenance details

Table S8. Software functionality

Topic	Actions undertaken
Option (or examples) on how to use another population dataset; e.g. country's official population grid	Option implemented; guidance provided in example comments
Option to use and report on population with demographic aspects --- e.g. Total population stratified by gender and age using population grid data for Catalunya and other areas in Europe	Implemented
OSMnx road network retrieved from Overpass API as 'current' road data, not historical; priority to find a way to use historical data for this	Retrieval matches publication date of configured PBF file
Intersection cleaning --- should be able to account for spatially varying urban typologies; currently a single tolerance parameter, which may not be optimal in all areas	Can optionally configure use of custom intersection data (e.g. official intersections)
Points of interest can be imported; but means to import public open space/areas of open space and other types of features (e.g. cleaned intersections) could be added too	Capability to use custom data for intersections implemented. Customisation of areas of open space remains future work
Interactive indicator web map to view/export indicators/comparisons at multiple scales with satellite basemap for built form and option to select different processed cities, locally served; later the Observatory	Capability to generate customised html choropleth maps including satellite basemap implemented; basic offline indicator atlas functionality implemented
Visual interface, like GBD, would be useful https://vizhub.healthdata.org/gbd-compare/	Basic comparison table implemented in web app; more advanced comparisons are future work

Table S9. Documentation and guidance

Topic	Actions undertaken
Recent changes have made the tool easier, and easier to understand	Positive feedback
Screenshots of results and terminal were useful	Positive feedback
Videos are going to help a lot of people	Video tutorials produced
Should have simple to follow directions; ideally this should be for a person who has some technical expertise but not a computer science expert	Detailed guidance provided via website, Jupyter notebook, GitHub and videos
User example: not an expert in programming, but can learn; interested to calculate indicators	Provided graphical interface as usage option, and guided Jupyter notebook example illustrating how to perform analyses and comparisons using code
Councils all have geospatial teams, these would be an example of the kind of users	Advice on users
Geospatial analysis is popular in urban planning and architecture, but code is a barrier to implementation ---- providing code examples lowers the barrier	Code examples provided along with graphical and low-code options
Scripted videos pitched at right level, broken into steps	Scripted videos provided as playlist
Provide a summary/motivation at start of videos to provide intuitive introduction to topic, explain where going	Took care to make videos clear and focused
Short video snippets; as software is updated, sections can be updated; Can be curated in a playlist; will make easier to translate	Playlist of videos curated
Intersperse video snippet examples into the web page directions	Videos included in website guidance materials
Guidance on protocol or processes for public communication	To be actioned as part of future work
More examples for incorporating GTFS data	To be actioned as part of future work
Visual way of understanding the steps may help usability	Simplified the process into steps, and provided visual examples in website and videos
Video could blur out example policy results	Did not include illustrative policy checklist examples in videos; Updated policy analysis for example city has now been implemented.
Some users will need a 5 minute video, others more detailed advice	Guidance provided using multiple media formats

Table S10. Validation

Topic	Actions undertaken
Guiding users to validate is tricky -- may take some expertise to evaluate whether data is good/problematic/correctly configured; validation not currently implemented, expected of users to do this, but currently we aren't guiding them	More guidance provided on website and in example Jupyter notebook; additional guidance and tools for validation to be implemented as future work
Broader conversation needed for validation, very important	Critical feedback
Could guide users to compare results with parts of the city they know to identify whether/where process is working as expected given local knowledge	Provided examples on website and Jupyter notebook for how to do this
Poor quality, non-existance of open data is a challenge; open street map is not always good for a lot of cities in Brazil. For bigger cities yes, but for smaller cities data is scarce. Is there a way to incentivise a growth of open data?; Some advice on improving OpenStreetMap and guidance on validation will be useful	Provided some options for using custom data. Initiated conversation with YouthMappers. Links to materials providing guidance on

	contributing to OpenStreetMap could be provided as future work.

Table S11. Reports – as they are currently

Topic	Actions undertaken
Report is easy to understand, has right amount of explanations	Positive feedback
File formats and reports are ideal, the outputs are usable	Positive feedback
PDF template for web distribution useful	Positive feedback
Current reports are the old style (old policy indicators, comparisons), so maybe hold off on showing this; should take out reporting functionality from software until design is updated	Reporting functionality retained; additionally, report templates have been updated, see below.
Could present current reports (ie. not yet updated policy indicator) as this is what our current report looks like but its going to be updated; Cities will want to have some sort of report, important to find some sort of balance	Reports have been updated, see below.
Need to settle on a template quickly, make some decisions	Implemented.
Users want different templates	Three official templates implemented. Capability to modify templates is included; could be streamlined with examples provided as part of future work.
Need to be feasible about what is achievable, but need decision about how to present findings. One report first, other things later	Official templates for policy and/or spatial indicators are available (three templates, with translations in multiple languages)

Table S12. Reports – as they could be implemented

Topic	Actions undertaken
PDF template for printing would be useful; printed copy better for some stakeholders as can share directly	Implemented
Template for presentation, e.g. landscape PDF or powerpoint	Possible future work
Combined spatial and policy reporting, and flexibility for one or the other	Implemented
Official templates	Implemented
Flexibility for reporting to different audiences - indicator selection/thematic reporting (see contra-argument re integrated planning)	Possible future work
All the 'Ds' are important, so not looking at other Ds is problematic; these are a complete package of interventions that audiences should be exposed to	Critical feedback
Thematic sections could be within report	Report contains thematic sections
Including Ds framework language in the reporting, highlighting key areas grouped under principles	Following discussion, the Ds framework is not referenced explicitly in the report
Ds framework page with diagram and explaining integrated planning	Could include as online materials in future work
Don't want report too long; that the report is succinct is a strength	Aiming for brevity in current updates
Flexible selection and granularity of indicator calculating and reporting, e.g. transport indicators by mode, different indicators may be relevant for different cities, e.g. Abu Dhabi and heat related indicators	Possible future work
Flexibility for reporting to different audiences - Accessible language, different language may be required for different professionals eg urban planners and public health practitioners require different approaches to communication	*Possible future work* (users can optionally customise spoken and/or domain

	language details; examples could be provided for how to do this in future)
More detail on local environmental/geography/infrastructure/policy context e.g. climate/weather, topography, do they have light rail etc	Included a customisable study region urban context box, and map with analytical region boundary and satellite basemap
Configurability of report sections e.g. in YAML configuration	Reports can be customised using region configuration file
Context map for each city -- picture before jumping into results, with info about built up area, locations of features	Implemented
Scorecards for overall city	Single page 'scorecards' in the style of the 25-city project are planned to be implemented as future work
Reporting for different areas, multiple scales i.e. more informative for urban masterplans and to guide urban development; Reports with summaries for regions within city; e.g. compare each sub-district/district (as configured) to the city average	Users can configure study regions (eg for specific neighbourhoods) and area scales of reporting to influence how these are summarised.
Reports with summaries for collection of cities being analysed within a country; e.g. compare each Spanish city to the average of Spanish cities	Capacity for custom comparison targets planned for future update.
Provide users with a conceptual report on Ds framework and importance/methods/references for included indicators	Basic analysis report implemented; *more detail to be provided as part of future work.* Methods and references remain as cited in Lancet Global Health Series on Urban Design, Transport and Health.
Capacity to communicate findings graphically very important. Visual representation important.	Critical feedback
User friendly outputs, very important	Critical feedback

Table S13. New indicators

**Topic**	**Actions undertaken**
Air pollution	Planning for inclusion in future update
Noise pollution	Possible inclusion in future update
Public transport system; include docking stations and cycling infrastructure in this; could disaggregate PT	Planning for inclusion in future update
Cyclability; Bike sharing systems; docking stations and linear infrastructure often in OpenStreetMap -- could have cycling working group to prototype	Initiated cycling indicator working group; *Planning for inclusion in future update*
Spatial composite measure with heatmap highlighting areas with need for interventions - currently have a walkability index, could have a Ds index	Example for generating an interactive walkability choropleth map (HTML file) provided, in addition to static walkability maps. *Additional composite indicators are possible future work.*

Table S14. Comparisons

Topic	Actions undertaken
Comparisons with region-specific sets of cities; e.g. Spain, Europe, Latin America, as relevant. Different cities may require different comparisons, not all fair -- eg w/ compact cities in Europe, a different reality. Need to select relevant comparison cities	Implemented and provided examples for using a simple 'compare' tool that users can generate basic comparisons with
Comparisons need to be relevant for policy makers	Critical feedback
Self-selected comparisons could lead to low ambition, gaming	Critical feedback
Comparisons should be determined by Observatory executive to push ambition	Critical feedback
Could remove comparisons to avoid subjective interpretations/poor comparisons -- cities against themselves	Comparisons (of specific indicators against targets, and other cities) were desired by users, so functionality was retained. *Additional guidance/references for meaningful comparisons could be future work.*
Longitudinal comparisons within city; evaluate impact of urban actions, how liveability was before and after urban intervention; this is what urban planners and practitioners demand; Health impact evaluation of interventions, e.g. Superblocks only evaluated using models, not using real world data	Implemented 'compare' tool for basic evaluation of impact (change relating to real or hypothetical differences). *Health impact evaluation modelling, e.g. using population attributable fractions could be future work.*
Threshold comparisons, e.g. percentage of population meeting or within some relevant threshold	Implemented. Additional/more stream-lined customisation could be future work.
Clear and easily calculated indicators are important for meaningful comparability; otherwise comparisons can be difficult due to differences in design guidelines, focuses, indicators and methods	Critical feedback
Performance ranking of districts/neighbourhoods (but see contra-argument to ranking)	Users can do this themselves using generated data (*not a core objective*)
Comparisons very important, but not as a league table; ranking can deter participation	Ranking has been avoided
Allow for users to generate within/between city comparisons for the cities they are analysing (eg. Spanish cities), but Observatory executive curates official comparisons using supplied data for the Observatory	Implemented
A collection of hundreds of cities participating in the 1000 Cities Challenge could be used to inform benchmark averages in future	Possible future work

Table S15. Policy review

Topic	Actions undertaken
Distinction should be made between 'recommended' and 'mandatory' policies in policy review; recommendations are often superseded and change, become out of date - argument to restrict to mandatory	Implemented as additional item in policy checklist tool

Participant study information sheet

Delivered online at https://healthysustainablecities.github.io/software_feedback

Invitation to participate in an action research software development study

An action research-based framework for practitioner-led software design has been conceived to create a tool to support participation in the 1000 cities challenge. Action research is a qualitative research method founded on strong ethical and moral principles concerning the delivery of tangible results from participant-led research which addresses real world problems (Stringer, 2021).

Stakeholders in the fields of urban planning and policy research and practice who share an interest in the project goals are invited to participate as co-researchers in the planning, design and evaluation of the software implementation through to the formal release of the open source software.

Why participate?

An increasing majority of the world's population reside in cities, however inequities of health, wealth and opportunities exacerbated by how we design and manage cities pose challenges not only for individual and population health and wellbeing, but also planetary sustainability.

An international collaborative group of built environment and physical activity researchers, the Global Healthy and Sustainable City Indicators Collaboration, seek to develop a tool to support evaluation and monitoring of policy and spatial indicators to support interventions promoting healthy, sustainable cities in diverse contexts globally. Following an initial scoping project for 25 cities (https://www.healthysustainablecities.org/publications), a '1000 city challenge' has been proposed (https://www.healthysustainablecities.org/1000cities).

Feedback gathered from potential users of the software will help guide the design of an open source tool to meet the needs of urban planning and health stakeholders who seek to benchmark and monitor plicy and spatial indicators for healthy and sustainable cities in diverse settings.

What does it involve?

Stakeholder participants will complete a short survey (linked below) and be engaged through optional monthly online focus group sessions and on-going opportunities for feedback via e-mail or GitHub in progressing the project through three key project phases: 1) Clarifying goals and elicitation of user requirements; 2) modelling a comprehensible and sustainable software architecture that meets the needs of domain experts; 3) on-going evaluation of prototype software, as stakeholder feature requests are progressively implemented according to consensus on user priorities. This will be achieved through iteration through cycles of observation, evaluation and implementation (or 'Look-Think-Act' (Stringer, 2021)) through to final delivery of the research objectives, as represented in Figure 1 below.

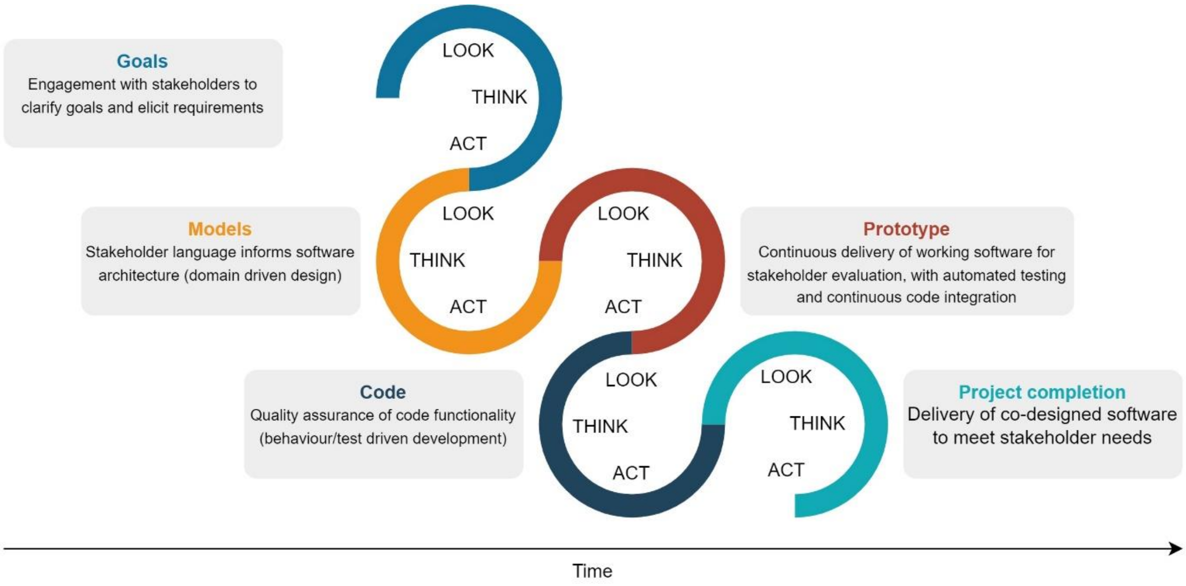

Figure 1. Software development envisaged through the framework of participatory action research

The study is anticipated to span 4 months from March 2022 to July 2023, although this may be extended as the criteria for successful realisation of working software for a formal release will be determined through the collaborative action research process.

No prior knowledge of software development is required of participants, whose engagement is predicated on interest and expertise in the intersection of urban planning, health and sustainability and the monitoring and evaluation of policy and spatial indicators to support these goals in specific local contexts. Participants with an interest in active contribution to coding may do so using the public facing GitHub project page which will be used for development. Community facing project contribution guidelines will be developed as part of the action research process.

To understand user cohort diversity, basic demographic data will be collected and summarised (e.g. age, sex, professional field(s), years of engagement in professional field(s), qualifications, city and primary languages of practice, prior engagement in the Global Healthy and Sustainable City Indicators Collaboration). This information will be used to contextualise aggregate feedback in a way that is respectful and professional, and with consent from participants. Participants will have the opportunity to review and clarify any records produced through the project to ensure their feedback and engagement with the project is accurately and fairly represented.

The research is not interested in sensitive or personal details, however participants can request anonymity in records and summaries produced through the course of the research; a random generated identifier will be used in such cases, with identity linkage securely stored.

What will the research outputs be?

The primary output is an open source software package (creative work/tool), which will be hosted and maintained on GitHub and RMIT Figshare. A journal article detailing the development process and usage of the software will be published in a Q1 journal, and presented on in at least one conference. Participants who have actively participated as co-researchers will be invited to be included as co-authors, as per the recommendations

of the International Committee of Medical Journal Editors. Additional journal articles presenting both cross-sectional and longitudinal analyses conducted using the software for the 1000 city challenge are envisaged as long term outcomes. The outputs arising from the usage of the software will include data, figures and PDF policy reports in multiple languages with broad scope for impact on policy and practice in city planning. The software is envisaged to encourage and facilitate the public dissemination of such research outputs, for example, through streamlining the process of archival in public repositories and indexing with persistent DOI identifiers.

Study details

Human research ethics approval to conduct an action research study was granted on 5 September 2022 by the RMIT University Design and Social Context (DSC) College Human Advisory Network (CHEAN). The project (#25552) was considered to be negligible/low risk, with an anticipated end date of 1 August 2024.

This research project is being conducted by Carl Higgs (PhD Candidate), Emeritus Professor Billie Giles-Corti, Dr Dhirendra Singh, Dr Sebastian Rodriguez and Dr Melanie Lowe..

For further information about the research and its outcomes please contact carl.higgs@rmit.edu.au. Should you have any concerns or questions about this research project, which you do not wish to discuss with the researchers listed in this document, then you may contact humanethics@rmit.edu.au.

Complete the survey and participate in the study

To participate, we ask that a short (5-10 minutes) baseline survey be completed, with optional further participation in feedback groups.

The Google Forms survey can be accessed here.

Thank you for your interest in participating in our research!

References

Stringer ET, Aragón AO (2021) Action Research, 5th ed. SAGE Publications, Thousand Oaks, CA

Evans E (2003) Domain-Driven Design: Tackling Complexity in the Heart of Software. Addison-Wesley

Farley D (2022) Modern Software Engineering: Doing What Works to Build Better Software Faster. Pearson

Liu S, Higgs C, Arundel J, Boeing G, Cerdera N, Moctezuma D, Cerin E, Adlakha D, Lowe M, Giles-Corti B (2021) A Generalized Framework for Measuring Pedestrian Accessibility around the World Using Open Data. Geographical Analysis. https://doi.org/10.1111/gean.12290

Higgs C, Alderton A, Rozek J, et al (2022) Policy-Relevant Spatial Indicators of Urban Liveability And Sustainability: Scaling From Local to Global. Urban Policy and Research. https://doi.org/10.1080/08111146.2022.2076215

Standards for Reporting Qualitative Research guidelines checklist responses

Qualitative reporting research guideline checklist based on: O'Brien, Bridget C. PhD; Harris, Ilene B. PhD; Beckman, Thomas J. MD; Reed, Darcy A. MD, MPH; Cook, David A. MD, MHPE. Standards for Reporting Qualitative Research: A Synthesis of Recommendations. Academic Medicine 89(9):p 1245-1251, September 2014. DOI: 10.1097/ACM.0000000000000388

Table S16. Standards for Reporting Qualitative Research guidelines checklist responses

#	Topic	Item response or page #
	**Title and abstract**	
1	Title	Provides a concise description of the nature and topic of the study, identifying collaborative approach to research
2	Abstract	Summarises key elements in a standard format (background, purpose, methods, results, conclusions)
	**Introduction**	
3	Problem formulation	Pages 2-3
4	Purpose	Page 3
	**Methods**	
5	Qualitative approach and research paradigm	Page 4
6	Researcher characteristics and reflexivity	Page 5 (the study executive themselves were considered one of three target groups of co-researchers), Pages 5-6 (we report the background characteristics of the involved co-researchers), Pages 14-15 we reflect on the limitations and strengths of our novel fusion of action research and modern software engineering practices. Through out the paper and supplementary material, in qualitative and quantitative analyses, we detail perspectives of the study executive as internal stakeholders and broader collaborative network as external stakeholders who were intended to reflect the broader group of users the software was to be designed for.
7	Context	Page 3
8	Sampling strategy	Page 5
9	Ethical issues pertaining to human subjects	Page 5, including details of ethics approval and link to study information (alse see Study information in Supplementary material, pages 21-22)
10	Data collection methods	Page 5, with further detail in supplementary material (supplementary pages 2-3, including study information on page 20)
11	Data collection instruments and technologies	Page 5 and supplementary material (supp. pages 2-3)
12	Units of study	Page 5
13	Data processing	Page 5 and supplementary material (supp. pages 2-3), also see accompanying code
14	Data analysis	Page 5 and supplementary material (supp. pages 2-3)
15	Techniques to enhance trustworthiness	Ethics approved action research methodology (page 5; supplementary material pages 20-22), critical engagement with open science (pages 3, 15-16) and open data (page 18)
	**Results**	
16	Synthesis and interpretation	Pages 6-12
17	Links to empirical data	Page 18
	**Discussion**	
18	Integration with prior work, implications, transferability and contribution(s) to the field	Pages 12-18
19	Limitations	Pages 14-18
	**Other**	
20	Conflicts of interest	Page 18
21	Funding	Page 18
